# Quantitative Evaluation of the Mechanical Risks Caused by Focal Cartilage Defects in the Knee

**DOI:** 10.1038/srep37538

**Published:** 2016-11-29

**Authors:** Mikko S. Venäläinen, Mika E. Mononen, Jari Salo, Lasse P. Räsänen, Jukka S. Jurvelin, Juha Töyräs, Tuomas Virén, Rami K. Korhonen

**Affiliations:** 1 Department of Applied Physics, University of Eastern Finland, POB 1627, FI-70211 Kuopio, Finland; 2 Cancer Center, Kuopio University Hospital, POB 100, FI-70029 KUH, Kuopio, Finland; 3 Department of Orthopaedics, Traumatology and Hand Surgery, Kuopio University Hospital, POB 100, FI-70029 KUH, Kuopio, Finland; 4 Hospital Mehiläinen, FI-00260, Helsinki, Finland; 5 Diagnostic Imaging Center, Kuopio University Hospital, POB 100, FI-70029 KUH, Kuopio, Finland

## Abstract

Focal cartilage lesions can proceed to severe osteoarthritis or remain unaltered even for years. A method to identify high risk defects would be of utmost importance to guide clinical decision making and to identify the patients that are at the highest risk for the onset and progression of osteoarthritis. Based on cone beam computed tomography arthrography, we present a novel computational model for evaluating changes in local mechanical responses around cartilage defects. Our model, based on data obtained from a human knee *in vivo*, demonstrated that the most substantial alterations around the defect, as compared to the intact tissue, were observed in minimum principal (compressive) strains and shear strains. Both strain values experienced up to 3-fold increase, exceeding levels previously associated with chondrocyte apoptosis and failure of collagen crosslinks. Furthermore, defects at the central regions of medial tibial cartilage with direct cartilage-cartilage contact were the most vulnerable to loading. Also locations under the meniscus experienced substantially increased minimum principal strains. We suggest that during knee joint loading particularly minimum principal and shear strains are increased above tissue failure limits around cartilage defects which might lead to osteoarthritis. However, this increase in strains is highly location-specific on the joint surface.

Focal cartilage defects are common findings in symptomatic knee joints^1^ and also known to be associated with the progressive degeneration of the tissue^2^,^3^. Focal defects can also be found in healthy subjects with no knee pain or evidence of radiographic osteoarthritis (OA)^3^. Although the progression of defects and development of knee OA are most likely multifactorial, articular defects change contact mechanics and mechanical response of tissue adjacent to a defect^4^,^5^,^6^,^7^,^8^. Furthermore, higher levels of contact stresses have been associated with the subsequent development of symptomatic knee OA^9^, thus indicating that the mechanical changes caused by cartilage defects may also critically contribute to the pathogenesis of OA.

It has been reported that under axial compression, contact stresses and stress gradients are elevated on cartilage surfaces adjacent to the rim of a focal defect^4^,^5^. As a consequence, tissue adjacent to the defect experiences increased deformation^6^ and local strains^7^,^8^. The elevated stresses and strains may reach levels that can induce cell death^10^ and matrix damage^11^, potentially leading to progressive degeneration in tissues adjacent to the defect.

Previously, the effects of focal defects on the mechanical environment of articular cartilage have been analyzed experimentally in *in vitro* systems^4^,^7^,^8^ and theoretically with whole joint finite element (FE) models including artificially introduced cartilage defects^12^,^13^,^14^. However, *in vivo* characterization of changes in the local mechanical response of cartilage in the vicinity of a clinically observed focal defect could improve understanding of the underlying biomechanical response and mechanobiology at the damaged region. Novel magnetic resonance imaging (MRI) and cone beam computed tomography (CBCT) arthrography techniques can help to estimate *in vivo* deformation of articular cartilage under simple loading configurations^15^,^16^. Particularly, CBCT arthrography has shown a great clinical potential for joint diagnostics, especially for the detection of cartilage defects^17^. Over clinical MRI, the benefits of CBCT arthrography include shorter imaging times, higher resolution and lower costs. However, computational techniques such as FE analysis based on clinical imaging may help to gain even deeper understanding of the development and progression of OA by providing more elaborate functional information from articular cartilage than imaging data alone^18^. Moreover, computational methods also allow the study of joint function under physiological loading conditions.

The aim of the present study was to develop a FE model of a knee joint with a focal cartilage defect based on high-resolution clinical imaging data and study the subsequent changes in local mechanical response of cartilage around the defect during the stance phase of gait. For this purpose, a symptomatic knee joint with a defect on the central tibial cartilage was imaged with CBCT arthrography. In addition, to investigate the effects of the observed defect on the surrounding tissue, similar defects in terms of the size and shape were modeled in various anatomical locations within the same articulating surface. Based on previous *in vitro* observations^7^,^8^, it was hypothesized that, in tissue adjacent to the defect, elevated levels of compressive and shear strains would be observed near the cartilage surface, specifically at central regions of the tissue involving direct cartilage-cartilage contact.

## Methods

### CBCT Arthrography

**Figure 1:**
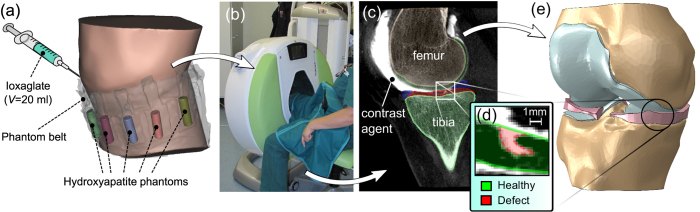
(**a**) Protocol for imaging and image processing. Prior to imaging, 20 ml of anionic ioxaglate was injected into the knee joint and five HA phantoms were placed around the knee. (**b**) After redistribution of contrast agent, the knee joint was imaged using a modern peripheral CBCT scanner. (**c**) A sagittal section of the knee showing contrast agent (bright area) and segmented tissue boundaries. (**d**) Close-up image of the segmented defect including a representation of the healthy tissue created by manually interpolating the intact tissue surface over the defect. (**e**) Assembly of solid geometries based on segmented tissues.

A 40-year old female volunteer with suspected knee joint injury was enrolled for a CBCT arthrographic study by a clinician’s referral and an informed consent was acquired from the patient. The study protocol was approved by the Research Ethics Committee of the Northern Savo Hospital District, Kuopio, Finland (Favourable Opinion No: 54/2011) and all experiments were carried out in accordance with the approved guidelines. The injured knee joint was imaged in sitting position using a modern CBCT scanner (Fig. 1b) (Verity, Planmed Oy, Helsinki, Finland) in a private clinic (Mehiläinen, Helsinki, Finland). Prior to imaging, 20 ml of anionic contrast agent (*q* = −1, *M* = 1269 g/mol, 320 mM, Hexabrix™, Mallinckrodt, Inc., St. Louis, MO, USA), diluted to half of its concentration using saline, was injected intra-articularly into the knee joint (Fig. 1a). Immediately after injection, the knee was flexed and extended gently with full range of motion to distribute the contrast agent evenly within the joint capsule. After redistribution of the contrast agent, five hydroxyapatite (HA) phantoms with known volumetric densities^19^ were placed around the knee at the height of proximal tibia. The knee was first imaged at 5 min after the injection to obtain a clear representation of surface geometries of cartilage and menisci (*i.e*. arthrographic image, Fig. 1c). Similarly as in a previous study^17^, a delayed image was acquired at 45 min after the injection of contrast agent. Here, due to lower concentration of contrast agent, the delayed image was utilized in the approximation of volumetric bone mineral densities (vBMDs) in order to minimize errors caused by beam hardening artefact often present in CBCT images. Tube voltage of 96 kV, tube current of 12 mA, and voxel size of 200 × 200 × 200 μm^3^ were applied during all imaging steps.

### CBCT image analysis

The contrast agent in CBCT arthrography provides a clear contrast between cartilaginous tissues and their surroundings making it applicable for simultaneous segmentation of cartilage, menisci and bone with a single imaging experiment^16^. All tissues in the model (cartilage, menisci, bone) were segmented using an open-source image segmentation software (Seg3D, http://www.sci.utah.edu/cibc-software/seg3d.html). Due to high resolution and the use of contrast agent in CBCT, a partial-thickness defect (surface area: 5.2 mm^2^, normalized defect depth: 63%, ICRS grade: 3) was clearly visible in the central part of the medial tibial cartilage (Fig. 1d). This defect was included in the segmented tibial cartilage by three-dimensional cropping around the lesion and applying Otsu’s thresholding method^22^ to separate the damaged cartilage surface from the synovial fluid. In addition to segmentation of the damaged tissue, a mask representing completely healthy tissue was created by manually interpolating the intact tissue surface over the defect. Other tissue areas were segmented manually or by using a combination of thresholding and morphological operations^17^.

### FE model construction

#### Model geometry

**Figure 2:**
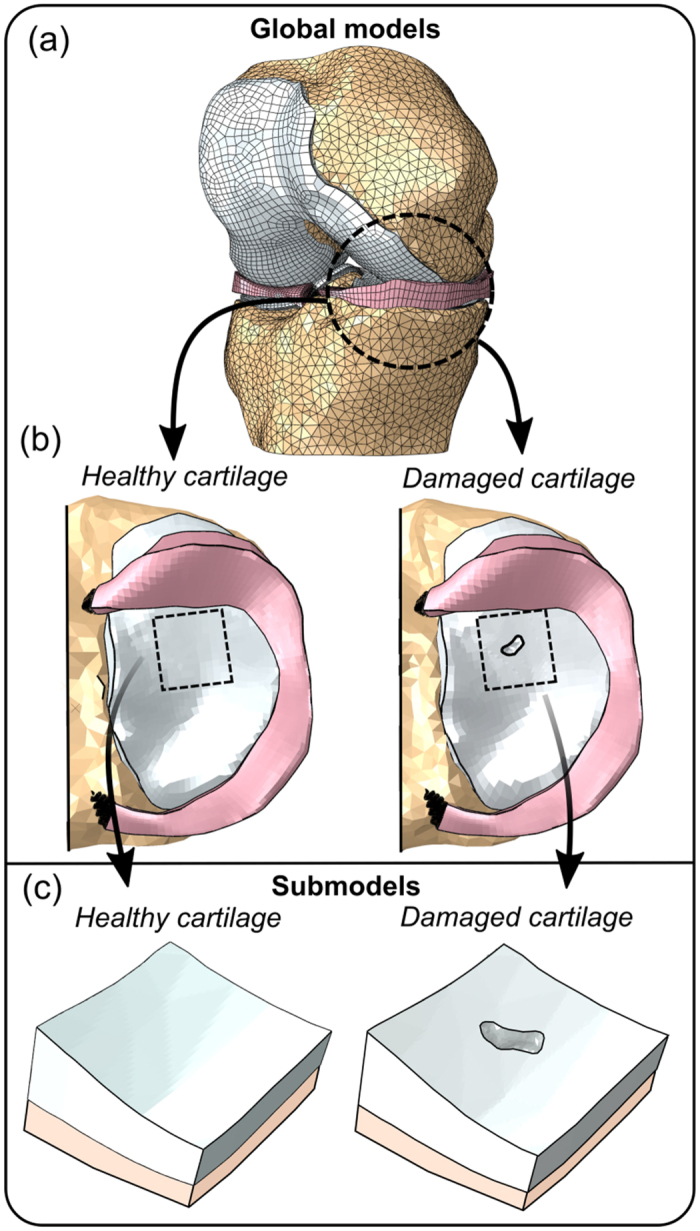
(**a**) FE representation of the knee with (**b**) fully intact and damaged tibial cartilage. (**c**) In order to improve the accuracy of the results, submodels of a 12 × 12 mm^2^ region of interest around the defect were created for both models.

For each segmented binary mask, a surface mesh was constructed and exported in stereolithography (STL) format for further post-processing in an advanced STL processing software (3-Matic v7.01, Materialise, Leuven, Belgium). Post-processing included minimization of small surface irregularities by smoothing and decreasing the number of tiny surface elements. Next, the processed surface meshes were converted into solid geometries (Fig. 1e) using a custom Matlab script (MATLAB R2014a, The MathWorks Inc., Natick, MA, USA) and imported into a commercial FE modeling package (Abaqus v6.13, Dassault Systèmes, Providence, RI, USA) for constructing the FE representation of the knee joint (Fig. 2a). In order to investigate the effect of the cartilage defect on the surrounding tissue, FE models with both intact tibial cartilage and the defect were created (Fig. 2b). The geometries of both models were identical, except inclusion/exclusion of the defect in the medial tibial cartilage.

#### Global models

For the accurate estimation of joint contact mechanics with minimum computational cost, the cartilage and menisci were meshed using linear hexahedral elements (C3D8(P), Fig. 2a). However, the defect site was meshed using tetrahedral elements to comply with the highly complex tissue geometry at that site. The bony tissues were modeled using linear tetrahedral elements. Element sizes of ~1.35 mm, ~1.0 mm, ~0.8 mm, and ~2.0 mm were used for femoral cartilage, tibial cartilage, menisci and bone, respectively. Since the global models were composed of up to 166,039 elements with computationally time demanding user defined material properties, submodeling approach was applied into the volume of the defect and its environment in order to result accurate and reliable outcomes of tissue stresses and strains around the defect area.

#### Submodels

For all global models, a rectangular region of interest (ROI) of approximately 12 × 12 mm^2^ was selected around the defect (Fig. 2b) and a submodel with higher mesh density was constructed for that region (Fig. 2c). In the ROI, there was at least 3 mm wide zone of intact cartilage tissue radially around the defect in the submodel. All submodels were meshed using linear tetrahedral elements thereby allowing the completion of the entire FE-analysis without excessive element distortion. Although this element type exhibits relatively low convergence with mesh refinement, the use of submodeling allowed the effective refinement of meshes to obtain accurate results within the ROI^23^. A mesh was considered to be dense enough when peak values of all analyzed parameters differed <2% from the solution obtained using a finer mesh. The final meshes of the submodels consisted of average element sizes of ~0.3 mm.

### Material properties

**Figure 3:**
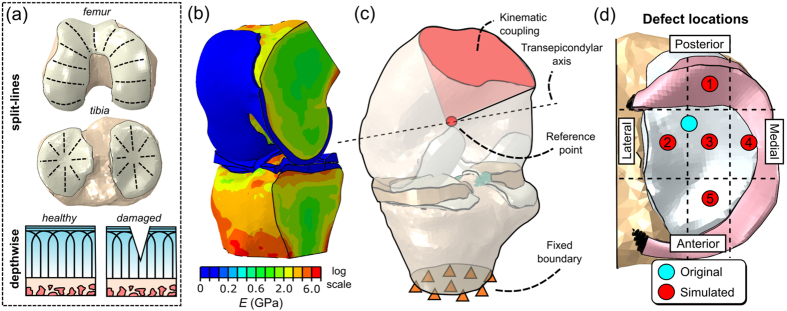
(**a**) Collagen architecture implemented for cartilage. (**b**) Inhomogeneous elastic properties of bone estimated from CBCT with the aid of HA phantoms. (**c**) FE model assembly showing the implementation of kinematic input (see^30^ for full description). (**d**) Original and alternative defect locations.

Articular cartilage and meniscus were modeled as a fibril-reinforced material (FRPVE)^24^,^25^,^26^ and as a linear elastic, transversely isotropic material^27^,^28^, respectively. Briefly, the FRPVE material is composed of a porohyperelastic non-fibrillar matrix, mimicking the mechanical behavior of tissue proteoglycans and fluid, and viscoelastic collagen fibrils. The collagen fibrils in the femoral and tibial cartilage tissues were oriented according to Benninghoff’s arcade-like structure^29^ folding into typical split-line patterns on the tissue surface (Fig. 3a)^26^. Meniscal attachments to the tibia were modeled using linear springs with a total spring constant of 350 N/mm at each meniscal horn^30^.

Similarly as in our previous study^31^, bone was modeled as a linear elastic, isotropic material with locally varying material properties. Young’s modulus, assigned to each element, was derived from the delayed image by calibrating the Hounsfield unit (HU) values to known volumetric bone mineral densities (vBMDs) and converting them to Young’s moduli with CT-derived density-elasticity relationships widely available in the literature^32^,^33^. Finally, the calculated Young’s moduli were mapped to each element with a custom Matlab script utilizing a mapping strategy based on numerical integration of Young’s modulus continuum field over the element’s volume (Fig. 3b)^31^,^34^,^35^.

### Simulations and boundary conditions

The effect of the cartilage defect on the mechanical response of adjacent tissue was simulated under loading conditions typical to the stance phase of gait. Similarly as in our previous studies^31^,^36^, experimental kinematic data of a single gait cycle (rotations and translations) and corresponding axial force data were obtained from literature^37^,^38^ and implemented as time-dependent boundary and load conditions into a reference point located at the center of transepicondylar axis of femur (Fig. 3c)^39^. All rotational degrees of freedom except varus-valgus rotation were coupled to the literature kinematics. In order to ensure sufficient cartilage-cartilage contact in both medial and lateral compartments during the entire gait cycle, varus-valgus rotation was allowed to be free similarly as earlier^31^,^40^. Medial-lateral and anterior-posterior translations were also coupled to the literature values, while the applied axial force was scaled using body weight of 71 kg.

Cartilage-bone interactions were modeled using tie constraints and the cartilage to cartilage and cartilage to menisci contact interactions were modeled using hard pressure-overclosure relationship and surface-to-surface discretization. The contact between articulating surfaces was assumed frictionless^41^. Since the permeability of cartilage is greater in the direction parallel rather than orthogonal to the orientation of the collagen fibers^42^, free fluid flow was allowed only through the inner defect surfaces but was restricted elsewhere.

In addition to the model with the original defect detected with CBCT arthrography, a total of five additional models with alternative locations for the defect (Fig. 3d) were created using a custom Matlab script. This was done by projecting nodes representing the surface geometry of the defect to intact tissue surface and computing local tissue penetration (normalized to local tissue thickness) for each node. Then, by moving projected nodes on the intact surface to the desired location, the geometry of the defect was reconstructed by evaluating local tissue thicknesses at the new location of each node and based on obtained values, recalculating *x*-, *y*- and *z*-coordinates with respect to tissue surface. By using this approach, it was possible to create alternative defects with constant relative tissue penetration depth and size projected on the cartilage surface. The model construction in additional defect cases followed the same protocol as with the original defect.

## Results

**Figure 4:**
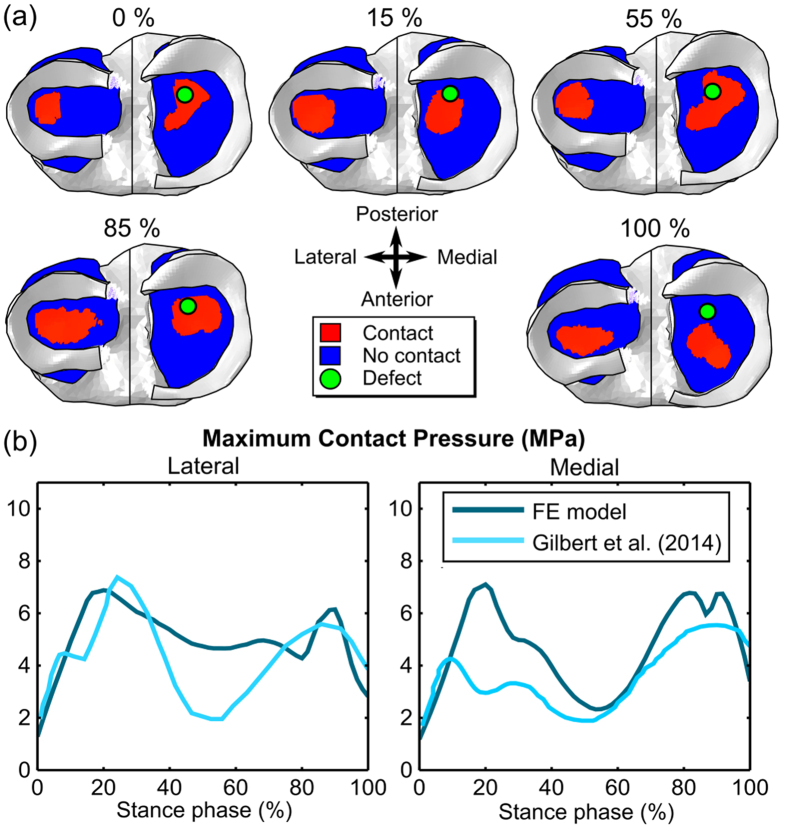
(**a**) Cartilage-cartilage contact patterns at different phases of stance. (**b**) Corresponding maximum contact pressures and experimental contact values from the literature^52^ as a function of stance phase.

**Figure 5:**
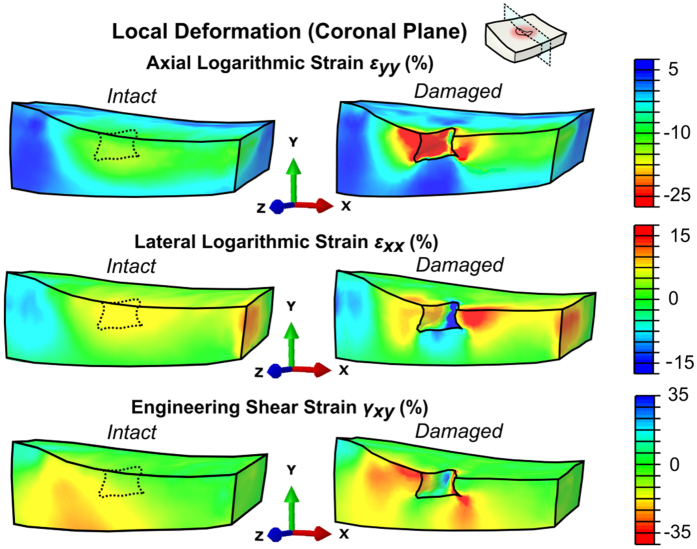
Axial and lateral logarithmic and engineering shear strains evaluated at 85% of the stance phase of gait (second peak load) for intact and damaged articular cartilage.

The distribution and magnitude of joint contact pressures varied according to the phase of stance and were consistent with the literature (Fig. 4a,b). As a result, time-varying differences between the models with intact and damaged cartilage were observed. Maximum differences in cartilage deformation were observed approximately at 85% of stance, *i.e*., during the second peak load of the stance phase (Fig. 5). At this time point, the defect was in a direct contact with the opposing femoral cartilage. The cartilage adjacent to the defect rim collapsed and stretched laterally, producing concentrations of shear strain both at the rim and tip of the defect (Fig. 5). However, opposite observations, *i.e.*, slight tensile axial strain and compressive lateral strain, as well as smaller shear strain, were made at the areas directly under the defect. Similar location-specific differences around the defect were also observed during other time points of the stance, however, they were smaller in amplitude.

**Figure 6:**
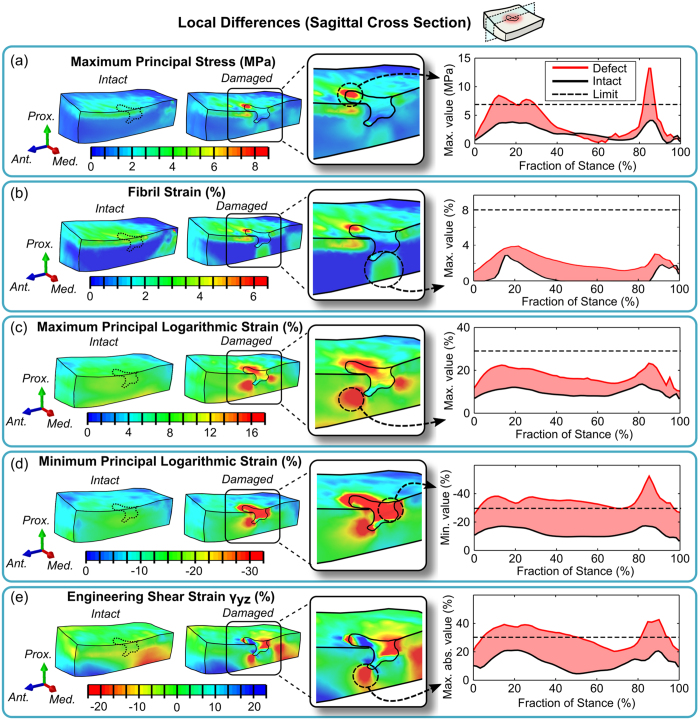
Local differences in (**a**) maximum principal stresses, (**b**) fibril strains, (**c**) maximum principal logarithmic strains, (**d**) minimum principal strains and (**e**) engineering shear strains at 85% of the stance phase of gait, the location of maximum differences between models with intact and damaged cartilage (middle) and the evolution of maximum values as a function of stance (right). The limits for potential tissue failure are indicated in graphs with dashed lines.

Failure analysis, based on the experimentally observed failure limits of stresses and strains, revealed potential failure areas in the adjacent tissues around the defect (Fig. 6). In terms of maximum principal stresses (Fig. 6a), a small area with elevated stress was observed at the defect rim. The absolute values at this area exceeded a suggested cartilage failure limit of 7 MPa^18^,^43^ during the peak loads of the stance (approximately at 10–30% and 80–90% of the stance). At other time points, the stresses were found to be well below the suggested failure limit. An area of elevated fibril strains was also observed at the same location, but the most predominant changes in fibril strains were found in deeper layers of cartilage (Fig. 6b). However, the absolute values of fibril strains were still relatively low and did not exceed a previously utilized threshold of 8%^44^ for collagen network damage at any time point of the stance. Approximately two-fold increase was observed in maximum principal strains (Fig. 6c) at the tip of the defect throughout the stance but the level of tensile strains was below the suggested failure of cartilage matrix, *i.e*., less than 30%^45^. The most substantial changes were found in minimum principal strains and shear strains which both clearly exceeded the failure limits of −30% and 32%, suggested earlier to induce chondrocyte apoptosis^46^ or failure of collagen crosslinks^47^, respectively (Fig. 6d,e). Both of these failure limits were exceeded from approximately 5% to 70% or 50% of the stance phase, respectively, and also at the second peak load (approximately at 70–97% and 80–95% of the stance, respectively).

**Figure 7:**
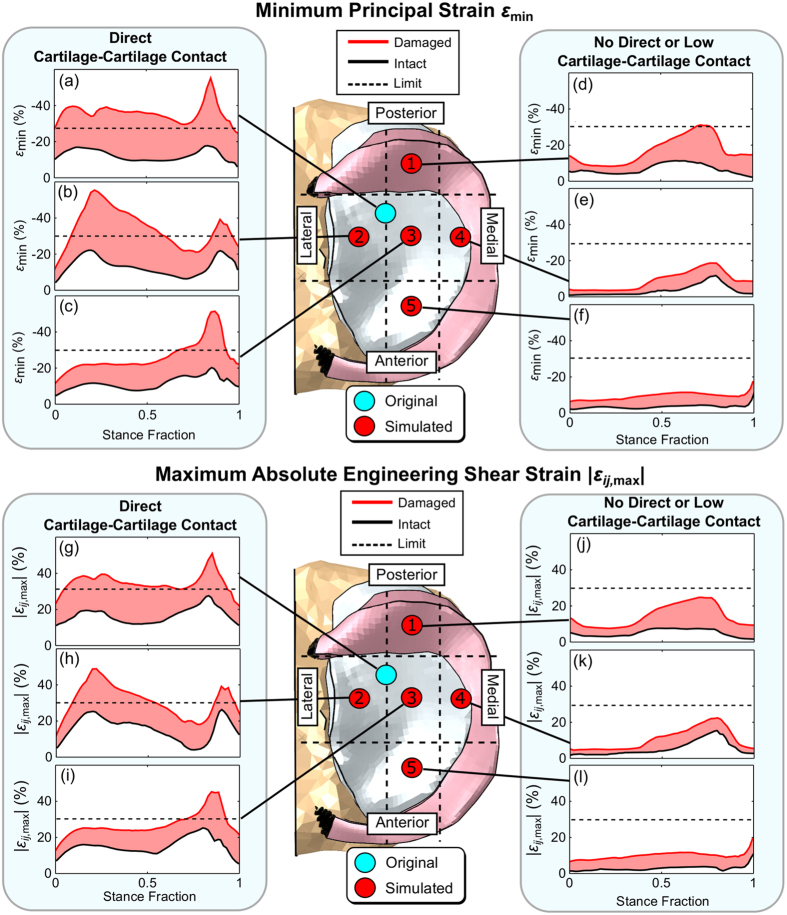
Maximum values of minimum principal and shear strains within 1 mm radius of the defect (here approximately the size of the defect location indicator) at different locations within medial tibial cartilage.

Defect location influenced substantially the local mechanical response of the adjacent cartilage (Fig. 7). In the areas with direct cartilage-cartilage contact, maximum values of both minimum principal and shear strains exceeded their suggested failure limits and their absolute values showed up to 3-fold increase depending on the phase of stance and location of the contact area (Fig. 7a–c,g–i). In general, the failure limits were exceeded at time points close to the peak loads. The failure thresholds were not exceeded at any location in the intact cartilage tissue (Fig. 7a–l). In the areas with no direct cartilage-cartilage contact (Fig. 7d–f,j–l), the suggested failure thresholds were not exceeded, except at approximately 75% of the stance. Then, with the defect in the posterior part of tibia under the meniscus, the minimum principal strains just reached the failure limit (Fig. 7d). Even though the thresholds were not exceeded elsewhere, still a noticeable increase (up to 8%) in the level of compressive and shear strains, as compared to the intact tissue, was observed throughout the stance phase due to gross deformation of the damaged tissue. In maximum principal stresses, maximum principal strains and fibril strains, only the maximum principal stress exceeded the corresponding failure limit at the defect rim in the alternative defect case no. 2 (data not shown).

## Discussion

In the present study, a FE model of a knee joint with a subject-specific focal cartilage defect was developed and the effects of the defects, as found in various different locations in medial tibial cartilage, on local mechanical responses of the tissue and potential cartilage failure were evaluated during the stance phase of gait. To our knowledge, this is the first time when a clinically observed cartilage defect was implemented into a whole knee joint FE model. The results obtained using the model were in accordance with previous *in vitro* studies examining the deformation behavior of isolated cartilage samples^7^,^8^. It was observed that the elevated levels of stresses and strains, especially during the peak loads of the stance phase (from 10% to 30% and from 80% to 90% of stance), could both indicate potential failure or further degeneration of the damaged cartilage tissue (Fig. 6). The most prominent changes, as compared to the intact tissue, included increased magnitudes of compressive and shear strains. They exceeded the suggested failure limits for major proportions of the stance phase. During those time periods, they reached levels that could induce either chondrocyte death^46^ or failure of collagen crosslinks^47^. Furthermore, the stress concentrations on the defect rim at peak loads could also imply the initiation of collagen matrix degeneration in the superficial layers of the tissue^18^,^43^. Exceeding the suggested failure limits of any of these parameters could lead to propagation of the defect and OA.

The key determinant for the changes in the corresponding local mechanical response of the tissue, due to a local tissue defect, was the location of the defect (Fig. 7). In the areas with a direct contact between the defect and opposing articulating surface (original and alternative defect cases 2 and 3), compressive strains exceeded a strain level previously associated with chondrocyte apoptosis^46^. Furthermore, it has been suggested that cell death, even in the form of apoptosis, appears to be linked to matrix degradation and further tissue damage in cartilage^46^,^48^, potentially contributing to the development of post-traumatic OA^49^. In addition to compressive strains, also shear strains were found to reach potentially harmful levels that could induce damage to collagen fibril network^47^ and thus lead to increased wear and fibrillation of the tissue. However, increased shear might also alter chondrocyte metabolism and increase release of nitric oxide associated with cell apoptosis^50^.

In the areas with no full direct cartilage-cartilage contact (alternative defect cases 1, 4, and 5), both compressive and shear strains increased over those in the intact tissue. Therefore, due to geometrical discontinuity introduced by the defect and gross tissue deformation, the risk for the progression of defect can still be elevated, although up to smaller extent, even outside the direct cartilage-cartilage contact. The most prominent changes in the local mechanical environment were observed with the submeniscal defect located at posterior parts of medial tibial cartilage where compressive strains barely reached the limit of −30% for chondrocyte apoptosis at the second peak load during gait. This indicates that the mechanical forces transmitted through the meniscus can create a challenging environment for submeniscal defects to heal. This kind of submeniscal cartilage damage is typically found especially under the posterior horn of lateral meniscus following an anterior cruciate ligament (ACL) injury and have been reported not be fully recovered even after ACL reconstruction^51^. Therefore, our results support also these observations.

The observed cartilage-cartilage contact pressures and locations were in close agreement with the experimentally measured contact pressures (Fig. 4b)^52^ and resulted in compressive deformation of the healthy tissue ranging from 10% to 18%. This behavior is comparable to previously determined *in vivo* cartilage deformations (ranging from 7% to 23%) during the stance phase of gait^53^. Different knee joint loading and geometry might lead to different contact patterns, leading possibly to different levels of stresses and strains between defects located in the medial and lateral compartment of the joint as well as between male and female subjects, depending on the body weight, kinematics, and the geometry and alignment of the knee joint. For this reason, we conducted additional analysis for another subject with different defect shape and knee joint loading. These results did not change conclusions drawn from results obtained from the original model (see supplementary Appendix A). Obviously differences can be seen in the values of strains and time periods when failure limits are exceeded. However, this only points out the importance of patient-specific analysis.

We included no patella, tendons, ligaments or muscle forces in the global models. These structures would certainly have an effect in models driven by forces and moments^54^. However, in the present computational model, the effects of all of these joint structures were considered in the knee joint motion and forces, similarly as has been done is several studies earlier^31^,^40^. When considering the aim of the current study, the inclusion of these features would have just caused unnecessary computational burden.

In the models, free fluid flow through cartilage boundaries was allowed on the inner defect surfaces since fluid flow along the collagen fiber direction is easier than perpendicular to the fibers^42^. For the same reason and due to the fast loading rate, free fluid flow was not allowed through other surfaces. This is supported by a study utilizing the same constitutive material model for cartilage as here showing that free fluid flow outside cartilage-cartilage contact had negligible effect on cartilage strains under creep loading^16^. Finally, we investigated both boundary conditions (free and sealed) for fluid flow on the inner defect surfaces but came to a conclusion that it can affect absolute values of stresses and strains slightly but does not change general conclusions drawn from those values.

Mechanical response of the cartilage tissue around a focal defect is likely to depend on defect size, shape, and local tissue composition. In the current study, effects of only one type of partial thickness cartilage defect were investigated. Thus, differences, as compared to intact tissue, might either be stronger or weaker when investigating different types of defects. Therefore, we carried out simulations with different defect sizes and were able to observe increases in minimum principal strains and shear strains, consistent with those obtained from the original knee joint model (see supplementary Appendix A). Obviously, the levels of stresses and strains can differ from those reported here which points out the importance of patient-specific defect segmentation and computational analysis. Furthermore, microstructure and composition of the adjacent tissue around the defect may eventually change, producing changes in the mechanical properties around the damage tissue. For example, it has been observed using an *ex vivo* model that fibrillation of the collagen network starts to develop and increase in the deep and superficial zones of cartilage adjacent to experimentally produced partial-thickness defects over a 4-week time period^55^. However, our aim was to evaluate the changes in the local mechanical environment immediately after the formation of the observed defect and thus we feel that our assumptions on material properties were justified. Therefore, any immediate changes in cartilage structure and composition were not considered here but could be taken into account in the future by carefully analyzing contrast agent diffusion within cartilage in delayed CBCT arthrography, *i.e*. by observing differences in contrast agent concentration between arthrographic and delayed images. The high concentration could highlight areas with decreased proteoglycan content or integrity of cartilage matrix^17^.

The present results, in terms of observed strains, were in accordance with experimental *in vitro* findings^7^,^8^. We suggest that cartilage defects, in particular those located in the central region of medial tibial cartilage, increase the risk for the development of knee OA. These observations are supported by a previous longitudinal study that reported more rapid progression of cartilage loss with defects located in the central region of the medial compartment as compared to anterior and posterior portions^56^. Nevertheless, the progression of cartilage defects has also been reported to be highly variable and may depend on several factors, including sex, age, and body mass index^57^.

In conclusion, computational modeling based on CBCT arthrography may provide a novel approach for diagnostics of cartilage defects. Further, the method may reveal risks for the development of post-traumatic OA particularly by recognizing locations with elevated levels of compressive and shear strains. When evaluating the mechanical response of cartilage with a defect in an individual, this approach could aid the clinical decision-making by identifying potentially harmful cartilage defects that, without an effective intervention, could promote progressive cartilage loss.

## Additional Information

**How to cite this article**: Venäläinen, M. S. *et al*. Quantitative Evaluation of the Mechanical Risks Caused by Focal Cartilage Defects in the Knee. *Sci. Rep*. **6**, 37538; doi: 10.1038/srep37538 (2016).

**Publisher’s note:** Springer Nature remains neutral with regard to jurisdictional claims in published maps and institutional affiliations.

## Supplementary Material

Supplementary Information
