# Publisher Correction: Quantitative Evaluation of the Mechanical Risks Caused by Focal Cartilage Defects in the Knee

**DOI:** 10.1038/s41598-019-41384-x

**Published:** 2019-04-04

**Authors:** Mikko S. Venäläinen, Mika E. Mononen, Jari Salo, Lasse P. Räsänen, Jukka S. Jurvelin, Juha Töyräs, Tuomas Virén, Rami K. Korhonen

**Affiliations:** 1 Department of Applied Physics, University of Eastern Finland, POB 1627, FI-70211 Kuopio, Finland; 2 Cancer Center, Kuopio University Hospital, POB 100, FI-70029 KUH Kuopio, Finland; 3 Department of Orthopaedics, Traumatology and Hand Surgery, Kuopio University Hospital, POB 100, FI-70029 KUH Kuopio, Finland; 4 Hospital Mehiläinen, FI-00260 Helsinki, Finland; 5 Diagnostic Imaging Center, Kuopio University Hospital, POB 100, FI-70029 KUH Kuopio, Finland

Correction to: *Scientific Reports* 10.1038/srep37538, published online 29 November 2016

Due to a typesetting error, this Article contains errors in the in-text citations. References 20 to 55 are incorrectly cited as References 22 to 57 respectively.

