# Supplementary material for: Quantitative Evaluation of the Mechanical Risks Caused by Focal Cartilage Defects in the Knee
Source: Sci Rep. 2016 Nov 29;6:37538. doi: 10.1038/srep37538 (PMC5126640; doi:10.1038/srep37538)
Supplement: Supplementary Information [file srep37538-s1.doc]

**APPENDIX A: Supplementary simulations**

For “*Quantitative Evaluation of the Mechanical Risks Caused by Focal Cartilage Defects in the Knee*” authored by Mikko S. Venäläinen, Mika E. Mononen, Jari Salo, Lasse P. Räsänen, Jukka S. Jurvelin, Juha Töyräs, Tuomas Virén and Rami K. Korhonen.

# Methods

## Models with modified defect sizes

In order to simulate the effect of the defect size on the mechanical response of cartilage, two additional FE models with reduced and increased defect sizes, as based on the original data, were constructed (Fig. A1). These models were created by modifying the binary mask segmented for the observed cartilage defect with three-dimensional morphological erosion and dilation until the depth of the defect reached 40% and 100% of the total tissue thickness. The surface areas of the reduced and enlarged defects were 1.21 mm^2^ and 26.4 mm^2^, respectively. After obtaining the geometries for modified defects, the FE models (both global models and submodels) were constructed as presented in the manuscript. For comparison, these models were also subjected to the same loading conditions as the original models and had the same material properties.


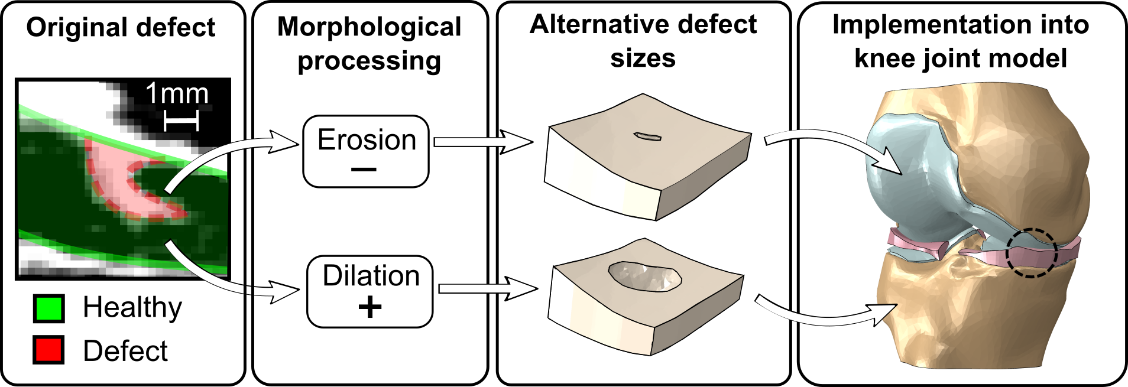


**Figure A1.** Workflow for creating knee joint models with modified defect sizes.

## Supplementary subject with a realistic defect

In addition to the knee studied in the manuscript, a defect detected in another patient using CBCT arthrography was implemented into an existing knee joint model utilized in our earlier study^18^. The defect, located approximately at the center of medial tibial cartilage (Fig. A2a), was segmented from the arthrographic CBCT image taken of a right knee of a 65-year old female volunteer. An informed consent was acquired from the patient and the study protocol was approved by the Research Ethics Committee of the Northern Savo Hospital District, Kuopio, Finland (Favourable Opinion No: 54/2011). All experiments were carried out in accordance with the approved guidelines. In addition, all imaging and segmentation steps utilized to obtain the geometry of the defect were carried out similarly as described in the manuscript.

In order to minimize testing time and optimization of the FE model, the observed defect was implemented into a functioning knee joint model (Fig. A2b) of a healthy 52-year old female subject (body weight 62.5 kg) based on information obtained from the osteoarthritis initiative database (OAI – hhtp://www.oai.ucsf.edu/). The defect was implemented into this model approximately at the same location to that observed in the arthrographic CT image. In order take into account any differences in surface curvature and tissue thickness, the defect was applied in the model by using the same Matlab script as for relocating the original defect to different locations within the joint. Finally, submodels for rectangular regions of interest around the defect were created for models both with healthy and damaged cartilage (Fig. A2c). For articular cartilage and menisci, same material properties were utilized as described in the manuscript. In this model bones were modeled as rigid.

The effect of the cartilage defect on the mechanical response of adjacent tissue was simulated under loading conditions typical to the stance phase of gait. However, the gait input was implemented differently compared to the model utilized in the manuscript and further information on implementing this particular gait load can be found in Mononen *et al.* (2016)^18^.


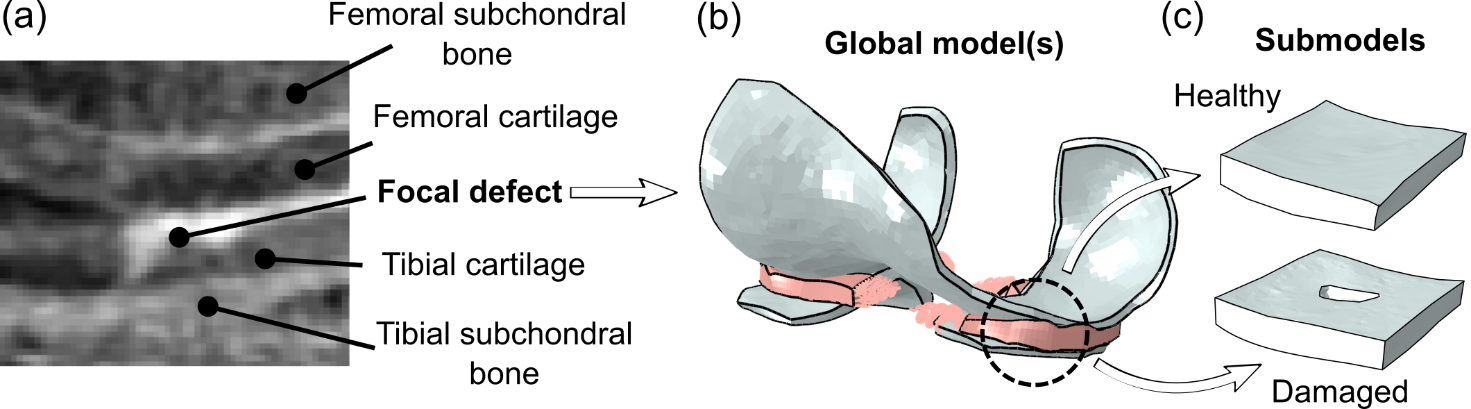


**Figure A2.** a) Focal defect observed in a knee joint of a 65-year old female volunteer using CBCT arthrography. b) FE knee joint model of a healthy 52-year old female subject based on data obtained from the osteoarthritis initiative database. c) Submodels of a region of interest surrounding the defect for both healthy and damaged cartilage.

# Results

## The effect of defect size

Similarly to that in the manuscript, the strains adjacent to the defect increased for both additional defect types and differences between healthy and damaged cartilage increased (Fig. A3). However, the size of the defect clearly affected the magnitude of the increase and higher values for strains were observed for the larger defect (Fig. A3b) than for the smaller defect (Fig. A3a). From the studied variables, only minimum principal strains and shear strains exceeded the failure limits described in the manuscript. For the larger defect, the failure limits were exceeded during the peak loads, *i.e.,* at time intervals from approximately 5% to 30% and from 70% to 90% of the total duration of the stance phase. However, around the smaller defect, the failure limits were exceeded for substantially shorter periods of time. In addition, shear strains did not exceed the failure limit during the first peak load.


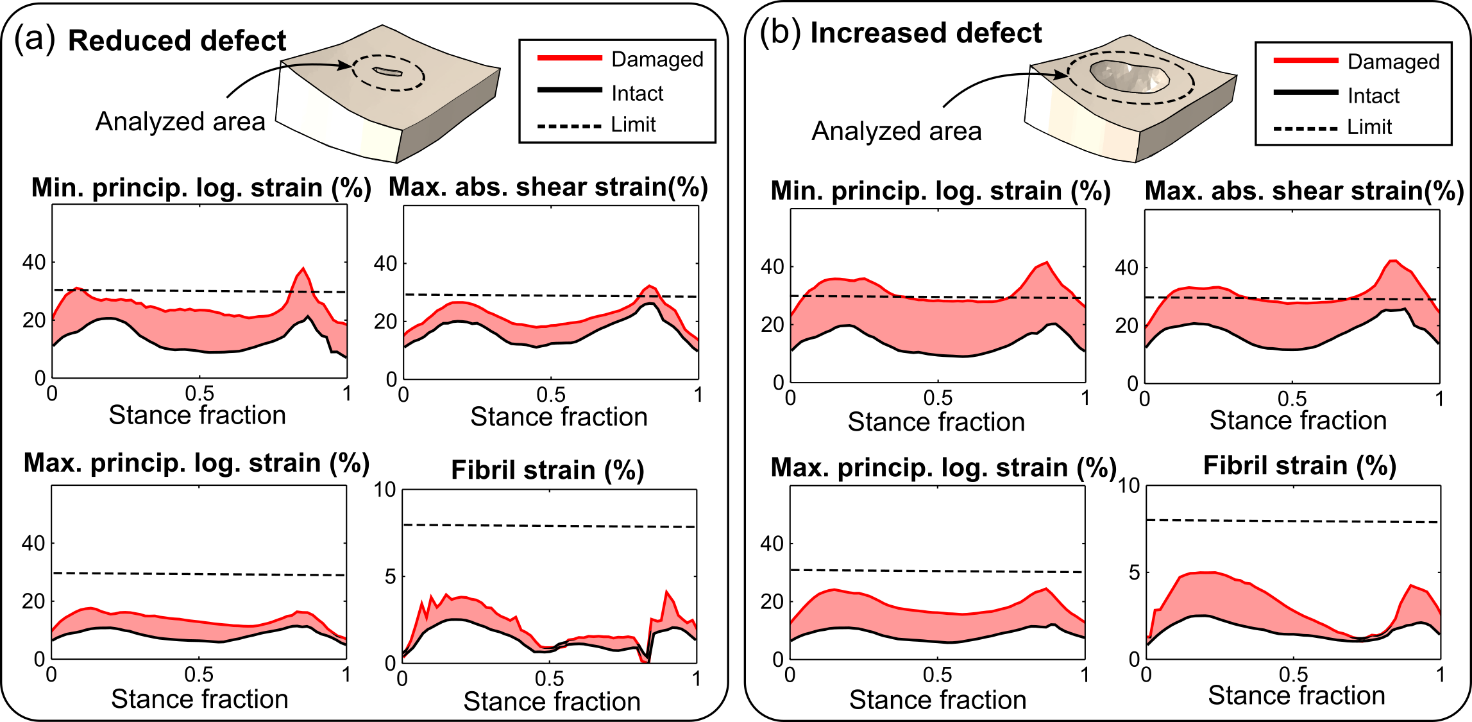


**Figure A3.** Maximum values of minimum and maximum principal logarithmic strains, shear strains and fibril strains within 1 mm radius of the defect for a) reduced defect and b) increased defect as compared to healthy tissue.

## Supplementary subject

As with all the studied defects, increases in compressive, tensile and shear strains were observed around the defect, as compared to healthy tissue (Fig. A4). Also, the minimum principal (compressive) strains (Fig. A4a) and shear strains (Fig. A4c) were found to exceed the corresponding failure limits at 50% of stance and remained above that level until approximately 90% of stance. The failure limits were exceeded briefly also during the first peak load, *i.e.* at approximately 20% of stance. Although a minor increase was observed also in maximum principal strains, the failure limit was not exceeded at any point of the stance phase.


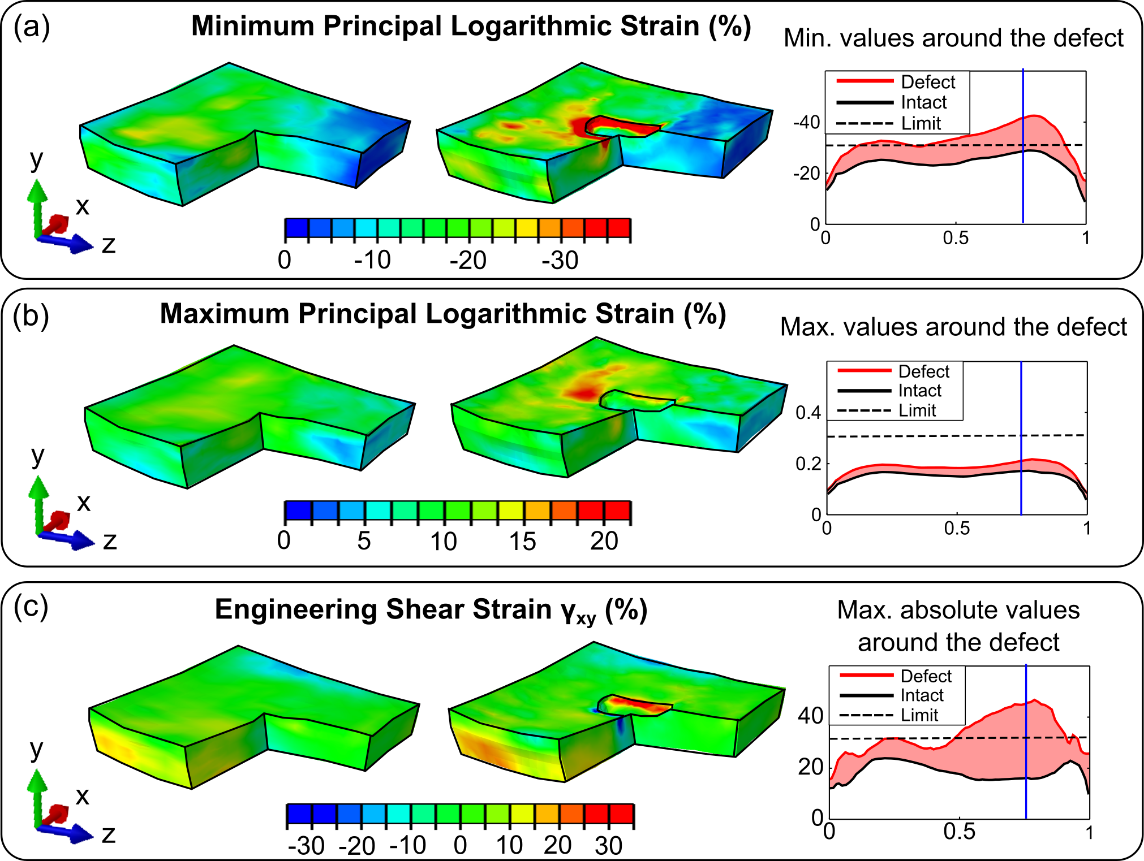


**Figure A4.** Local differences in a) minimum principal strains, b) maximum principal strains and c) engineering shear strains at 75% of the stance phase of gait and peak values as a function of stance fraction for subject no. 2.

# References

18. Mononen, M.E., Tanska, P., Isaksson, H. & Korhonen, R.K. A Novel Method to Simulate the Progression of Collagen Degeneration of Cartilage in the Knee: Data from the Osteoarthritis Initiative. *Sci Rep* **6**, 21415 (2016).
